# Phase Morphology and Mechanical Properties of Super-Tough PLLA/TPE/EMA-GMA Ternary Blends

**DOI:** 10.3390/polym16020192

**Published:** 2024-01-09

**Authors:** Martin Boruvka, Roman Base, Jan Novak, Pavel Brdlik, Lubos Behalek, Chakaphan Ngaowthong

**Affiliations:** 1 Department of Engineering Technology, Faculty of Mechanical Engineering, Technical University of Liberec, Studenstka 2, 461 17 Liberec, Czech Republic; roman.base@tul.cz (R.B.); jan.novak@tul.cz (J.N.); pavel.brdlik@tul.cz (P.B.); lubos.behalek@tul.cz (L.B.); 2 Department of Agricultural Engineering for Industry, Faculty of Industrial Technology and Management, King Mongkut’s University of Technology North Bangkok Prachinburi Campus, 29 Moo 6, Tumbon Noenhom, Muang 25230, Prachinburi, Thailand; chakaphan.n@fitm.kmutnb.ac.th

**Keywords:** multicomponent blends, super tough, co-continuous, poly(l-lactic acid) (PLLA), biodegradable thermoplastic polyester elastomer (TPE), ethylene-methyl acrylate-glycidyl methacrylate (EMA-GMA)

## Abstract

The inherent brittleness of poly(lactic acid) (PLA) limits its use in a wider range of applications that require plastic deformation at higher stress levels. To overcome this, a series of poly(l-lactic acid) (PLLA)/biodegradable thermoplastic polyester elastomer (TPE) blends and their ternary blends with an ethylene-methyl acrylate-glycidyl methacrylate (EMA-GMA) copolymer as a compatibilizer were prepared via melt blending to improve the poor impact strength and low ductility of PLAs. The thermal behavior, crystallinity, and miscibility of the binary and ternary blends were analyzed by differential scanning calorimetry (DSC). Tensile tests revealed a brittle–ductile transition when the binary PLLA/20TPE blend was compatibilized by 8.6 wt. % EMA-GMA, and the elongation at break increased from 10.9% to 227%. The “super tough” behavior of the PLLA/30TPE/12.9EMA-GMA ternary blend with the incomplete break and notched impact strength of 89.2 kJ∙m^−2^ was observed at an ambient temperature (23 °C). In addition, unnotched PLLA/40TPE samples showed a tremendous improvement in crack initiation resistance at sub-zero test conditions (−40 °C) with an impact strength of 178.1 kJ∙m^−2^. Morphological observation by scanning electron microscopy (SEM) indicates that EMA-GMA is preferentially located at the PLLA/TPE interphase, where it is partially incorporated into the matrix and partially encapsulates the TPE. The excellent combination of good interfacial adhesion, debonding cavitation, and subsequent matrix shear yielding worked synergistically with the phase transition from sea–island to co-continuous morphology to form an interesting super-toughening mechanism.

## 1. Introduction

The leakage of plastics into the environment, dwindling fossil resources, and high CO_2_ emissions are key issues surrounding today’s polymer products [1,2]. Despite current efforts, global plastic production continues to grow [3] and the material circularity of plastics remains stagnant at a very low level [4]. This situation is not solely due to bad consumer behavior and poor waste management. The root of the problem lies primarily in multinational corporations across the petrochemical value chain, which are trying to contain environmental crises, and the circular economy serves them as a catalyst for market expansion [5,6,7]. A recent review [8] of initiatives addressing plastic circularity reveals that there are barriers limiting the implementation to work at scale, such as financial, organizational, technological, policy-related, market-based, and social. In addition, most initiatives focus mainly on recycling, with much less attention paid to changes in plastic production, consumption, and waste prevention. The environmental benefits of improved recycling rates may be outweighed by the additional impacts of increased consumption. The use of secondary (recycled) materials then poses a significant challenge to increased recycling rates [9]. So far, the circular economy for plastics appears to be falling short of the United Nations (UN) Sustainable Development Goals (SDGs), particularly in categories such as responsible production and consumption (SDG 12) and climate change prevention (SDG 13) [10].

The implementation of bioplastics can offer improved circularity through the utilization of renewable resources, the introduction of new degradation/recycling pathways, and a lower carbon footprint [11]. However, these benefits are highly dependent on several factors (material source, chemical composition, manufacturing process, end-of-life scenario, etc.) and all of these need to be evaluated over the entire product life cycle [12]. Tools such as a comparative Life Cycle Assessment (LCA) to elucidate the environmental benefits of bio-based over fossil-based polymers must be used [13]. However, a fair comparison will hardly be possible if there is no equal footing. The same scrutiny and transparency that is demanded in new biobased products shall be applied vice versa to the petrochemical industry [14]. On the other hand, the challenges of bioplastics implementation, such as technological advances in biorefinery processes to increase efficiency and the supply of second-generation building blocks in a cost-competitive and sustainable manner, must be overcome. Future taxation of non-biobased materials will further drive demand for bioplastics, and environmental sustainability will be met with financial sustainability [15].

Despite these efforts, only a few bio-based or biodegradable polymers have successfully made their way to the market. Poly(lactic acid) or polylactide (PLA) is one of these biopolymers that has been extensively studied and produced on a large industrial scale [16]. Poly(d-lactic acid) and poly(l-lactic acid) represent two active optical PLA isomers, where the polymerization process, crystallization, and melting properties are highly dependent on the purity of the isomer [17]. PLA belongs to the family of aliphatic thermoplastic polyesters, has relatively good mechanical properties, and is both bio-based and biodegradable. Although its tensile strength and Young’s modulus are high, its inherent brittleness, which results in poor impact resistance and low ductility, limits its use in many industrial and medical applications that require plastic deformation at higher stress levels [18,19]. Over the past two decades, several strategies have been proposed to improve the properties of PLA. These include the use of plasticizers, copolymerization, blending with other polymers, the use of different reinforcements, nucleation additives, and many others [20,21,22].

Among these, polymer blending with carefully selected components is a convenient and cost-effective method to improve the toughness of PLA [23]. The blending of PLA with non-biodegradable polymers, usually derived from petroleum resources, has been adopted primarily to increase the bio-based content of these blends. The main polymer families that have been blended are polyolefins (PE [24,25,26,27] and PP [28,29,30]), vinyl copolymers (EVA [31,32,33,34] and EVOH [35,36,37]), styrene copolymers (ABS [38,39,40,41]), and various elastomers (PU [42,43,44,45,46] and TPEE [47,48,49]) or rubbers [50,51,52,53,54]. Given that utilizing petroleum-based substances to enhance PLA somewhat undermines sustainability, there has been growing attention on toughening PLA through renewable polymers like polyamide 11 (PA11) [55,56,57] or flexible copolymers such as polyether block amide (PEBA) [58,59,60]. In addition, blends of PLA with other biodegradable polymers are commonly pursued to both maintain biodegradability/compostability and reduce the risk of microplastic pollution. These represent polymers such as poly(ε-caprolactone) (PCL) [61,62,63,64,65], the polyhydroxyalkanoate (PHA) family [66,67,68], poly(butylene succinate) (PBS) [69,70,71,72], and poly(butylene adipate-co-terephthalate) (PBAT) [73,74,75,76,77].

Over the last few decades, several durable multicomponent PLA-based blends have been developed using reactive copolymers to enhance the phase morphology in a way that provides moderate stiffness and sufficient toughness. Approaches such as in situ reactive compatibilization and dynamic vulcanization have been employed in these efforts. These methods enhance interfacial strength by promoting chemical reactions between the blend components, creating robust stress transfer bridges [78]. The resulting blends, with their significant improvements in impact toughness, are often referred to as “super-toughened” PLA [79]. Wu [80] initially introduced the term “super tough” for ease of reference, indicating polymer blends with a notched impact strength exceeding 530 J∙m^−1^ (energy lost per unit width, North American standard) or 53 kJ∙m^−2^ (energy lost per unit cross-sectional area, European standard), depending on the dimensions of the sample.

Han et al. [60] prepared immiscible PLA blends containing up to 30 wt. % poly(ethylene oxide-b-amide-12) (PEBA). For blends containing 30% PEBA, the maximum notched impact strength of 60.5 kJ∙m^−2^ has been achieved, indicating a significant toughening effect. Zhang et al. [81] prepared PLA-based ternary blends with various contents and grades (Pebax Rnew) of a renewable poly(ether-b-amide) elastomeric copolymer (PEBA), which were compatibilized using different contents of ethylene-methyl acrylate-glycidyl methacrylate (EMA-GMA). Partial impact break behavior with a hinge, which was accompanied by a tremendous increase in the notched impact strength, was observed in the case of the PLA/EMA-GMA/PEBA (70/20/10) blend with a value of 410 J∙m^−1^. Feng and Ye [45] prepared a partially miscible system where was PLA toughened by increasing the content of a biocompatible thermoplastic polyurethane (TPU) elastomer with high strength and toughness. Blends with 30 wt. % TPU showed a bicontinuous phase structure with good interface compatibility and a notched impact strength of 58 kJ∙m^−2^. Liu et al. [82] studied the influence of poly-L-lactide (PLLA) melt blending with different contents of polyester-based TPU and poly-D-lactide (PDLA). Due to the stereocomplex crystallite formation between PLLA/PDLA, blends undergo a sharp brittle-ductile transition in impact toughness (from 9.6 kJ∙m^−2^ to 63.2 kJ∙m^−2^) when TPU content increases from 10 wt. % to 15 wt. %, where a morphological transition from a sea-island structure to a network-like structure occurs. In-mold annealed (i.e., 0.1 and 3 min, 130 °C) PLLA/15TPU/15PDLA samples (i.e., amorphous and highly crystalline) were studied to investigate the influence of the matrix crystallinity state on impact properties and thermal stability. Amorphous samples showed higher notched Izod impact strength (~75 kJ∙m^−2^). On the other hand, for highly crystalline samples, significantly higher heat resistance has been observed when compared to amorphous ones. The value of the storage modulus (G′) at 90 °C increased from 102.2 to 277.7 MPa. Dai et al. [83] investigated the same material systems based on PLLA blends with various contents of polyester-based TPU and PDLA. However, they used only small amounts (0.5–5 wt. %) of PDLA. They found that for PLLA/25TPU/xPDLA blends, notched Izod impact strength increases with higher PDLA content when the matrix is amorphous and decreases when the matrix is crystalline. Wu et al. [84] in their study prepared super-tough PLA/PBAT multicomponent blends using reactive melt-blending with EMA-GMA as a compatibilizer. When the content of EMA-GMA reached 8 wt. % (PLA/10PBAT/8EMA-GMA), a tremendous increase in the notched impact strength (45.2 kJ∙m^−2^) was observed. A further increase in EMA-GMA increased the notched impact strength of multicomponent blends by up to 61.9 kJ∙m^−2^, which is nearly 13 times higher than the value for the uncompatibilized PLA/10PBAT blend. However, a further decrease in the impact strength of multicomponent blends has been observed with the incorporation of 20 wt. % EMA-GMA. More recently, Wei et al. [85] demonstrated the effectiveness of a poly(styrene-co-glycidyl methacrylate) (SG)-based compatibilizer with high glycidyl methacrylate (GMA) content (45 wt. %). The best compatibilization occurs with the pre-grafted reactive compatibilizer of SG-g-PBAT (S1B1) with the SG-to-PBAT weight ratio of 1:2. The compatibilized PLLA/PBAT/SG (50/50/3) blend has co-continuous phase morphology and exhibited extremely high Charpy notched impact strength of 90.2 kJ∙m^−2^ and tensile strength of 33.3 MPa.

As discussed above, numerous papers have investigated the structure and composition of advanced polymer blends with their unique properties. In addition, a number of researchers have prepared multi-component PLA-based systems to achieve better performance, particularly in terms of mechanical properties. However, there has been no systematic study comparing compatibilized and non-compatibilized PLA blends in terms of their fracture behavior at ambient (23 °C) and low temperatures (−40 °C). The aim of our work was to investigate the relationship between structure/morphology evolution, toughening mechanisms, and brittle–ductile transitions at different temperatures. The systematic evaluation of crystallization behavior, fracture morphology, and mechanical performance was carried out to verify the superiority of the compatibilized blends and their potential use in durable applications.

## 2. Materials and Methods

### 2.1. Materials

Commercially available poly(l-lactic acid) (PLLA; Luminy L130) with an optical purity of ≥99% L-isomer, weight-average molecular weight (Mw) of 170 kg∙mol^−1^, and a polydispersity index (PI) of 1.95 was purchased from TotalEnergies Corbion (Gorinchem, Netherlands). A biodegradable thermoplastic polyester elastomer (TPE; NP EL 208-65A) was supplied by NaturePlast (Mondeville, France). A random copolymer of ethylene-methyl acrylate-glycidyl methacrylate (EMA-GMA) containing 8 wt. % of glycidyl methacrylate and 25 wt. % of methyl acrylate in pellet form was purchased from Merck (Darmstadt, Germany).

### 2.2. Preparation of Blends

Prior to melt blending, PLLA and TPE granules were oven-dried at 80 °C for 12 h under vacuum, and the same parameters were applied to EMA-GMA at 40 °C. Neat PLLA, PLLA/TPE binary blends, and compatibilized PLLA/TPE/EMA-GMA ternary blends were then melt-blended using an MC 15 HT microcompounder from Xplore (Sittard, The Netherlands). The PLLA/TPE/EMA-GMA ternary blends were designed to maintain a constant TPE/EMA-GMA ratio of 70/30 wt. % in the final sample compositions with PLLA. The resulting blends and their compositions are shown in Table 1. Each sample was melt-blended at 180 °C for 3 min using a built-in recirculation channel with a pair of co-rotating conical screws set at 100 rpm. The recirculation valve was then switched, and the homogenized melt was transferred to the pre-heated (180 °C) portable cylinder of the IM12 injection molding machine from Xplore (Sittard, Netherlands). Due to the fact that injection molds without a cooling system are regularly heated to a specific temperature after several cycles, we chose to preheat both molds to 50 °C to avoid differences in crystallinity. Standardized dumb-bell tensile test specimens (1B) according to ISO 178 and impact test specimens (80 × 10 × 4 mm) according to ISO 180 were prepared and released immediately after the injection molding cycle.

### 2.3. Differential Scanning Calorimetry (DSC)

The non-isothermal crystallization behaviors of neat PLLA, PLLA/TPE binary blends, and compatibilized PLLA/TPE/EMA-GMA ternary blends were investigated using DSC 1/700 from Mettler Toledo (Greifensee, Switzerland). Samples for measurements were prepared using the RM 2255 from Leica (Wetzlar, Germany) microtome from the middle part of impact specimen cross-sections (8 ± 0.5 mg). For each configuration, 2 samples were used, and the average values are shown. First, samples were heated at a rate of 10 °C∙min^−1^ from 0 °C to 200 °C, then samples were held isothermally at 200 °C for 3 min to eliminate thermal history. Samples were then cooled back to 0 °C at a cooling rate of 10 °C∙min^−1^ to observe melt crystallization. A second heating cycle from 0 °C to 200 °C at a heating rate of 10 °C∙min^−1^ was carried out to study the influence of blend compositions on crystallization without a thermal processing history. The following were recorded from the second heating phase: glass transition temperature (T_g_), cold crystallization temperature (T_cc_) and enthalpy (ΔH_cc_), pre-melting recrystallization temperature (T_rc_) and enthalpy (ΔH_rc_), and homocrystallite melting temperature (T_hm_) and enthalpy (ΔH_hm_). Some samples showed double melting peak behavior (**T_hm_**_α′_). Melt crystallization temperatures (T_mc1_, T_mc2_) and enthalpies (ΔH_mc1_, ΔH_mc2_) were recorded from the cooling phase. The parameters of the above temperatures and enthalpies were taken as the peak temperatures and the areas of the melting endotherms and crystallization exotherms, respectively. The crystallinity degree (χc) of PLLA in the blends was calculated as follows [86]:

(1) $$\chi_{c}=\frac{{∆H}_{hm}-{∆H}_{cc}{-∆H}_{rc}}{{∆H}_{hm}^{0}\cdot W_{m}}\cdot 100\left[\%\right]$$

where Δ*H*^0^_hm_ is the melting enthalpy of 100% crystallized PLLA (106 J∙g^−1^) [87] and *W*_m_ is the weight fraction of PLLA.

### 2.4. Mechanical Tests

All the samples were conditioned in a KSP from Teseco (Kostelec nad Orlicí, Czech Republic) climatic chamber according to ISO 291 at 23 °C and 50% relative humidity for 4 days prior to testing.

Tensile strength (σ_m_), elongation at break (ε_tb_), and Young’s modulus (E_t_) were measured using the TIRA test 2300 from Tira (Schalkau, Germany) universal testing machine equipped with a load cell of 10 kN and extensometer MFX 500-B from Mess & Feinwerktechnik GmbH (Velbert, Germany). Measurements were performed according to the ISO 527-1 standard [88]. Tensile strength and elongation at break measurements were performed at a crosshead speed of 5 mm∙min^−1^ and Young’s modulus at a crosshead speed of 1 mm∙min^−1^. Each batch of 1B-type dumbbell specimens was subjected to 10 repetitive tests under an ambient temperature of 23 °C. Average values with standard deviations are reported.

Charpy impact strength (a_cU_) was measured using a Zwick HIT50P from Zwick/Roell (Ulm, Germany) testing machine according to ISO 179-1/1eU standard [89]. A pendulum with a nominal energy of 50 J for an impact velocity of 3.8 m∙s^−1^ was used. Unnotched samples (80 × 10 × 4 mm) were used, and each batch was subjected to 10 repetitive tests under an ambient temperature of 23 °C and below zero temperature at −40 °C. Charpy notched impact strength (a_cA_) according to the ISO 179-1/1eA standard [89] was measured using A-type notched samples under the same conditions and on the same equipment.

### 2.5. Scanning Electron Microscopy (SEM)

The morphology of fractured surfaces was examined by field emission scanning electron microscopy (FE-SEM) TESCAN MIRA 3 from Tescan (Brno, Czech Republic) with an accelerated voltage of 5 kV. Notched and unnotched samples were taken from the impact testing measurements described above. Fractured surfaces were coated with 2 nm of platinum/palladium (Pt/Pd) alloy (80/20) using a sputter coater LEICA EM ACE200 from Leica (Wetzlar, Germany).

## 3. Results and Discussion

### 3.1. Thermal and Crystallization Behavior

**Figure 1:**
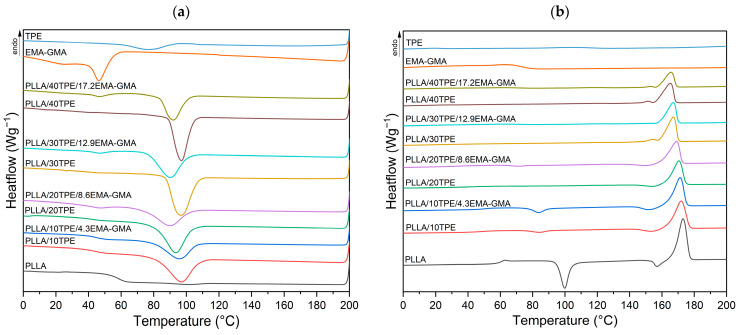
DSC curves obtained during the cooling (**a**) and second heating (**b**) at a rate of 10 °C∙min^−1^.

In order to draw clear conclusions about the mechanism involved in the improved toughness, it is essential to investigate the thermal properties and crystallinity of the blends. Of particular importance is the crystallization behavior of the main PLLA phase. The detailed results of the DSC analysis and the calculated values of the degrees of crystallinity are summarized in Table 2. Figure 1a represents the cooling phase where the crystallization curves of the individual blend components (PLLA, TPE, and EMA-GMA) and uncompatibilized (PLLA/TPE) and compatibilized (PLLA/TPE/EMA-GMA) blends are shown. Melt crystallization curves were recorded at a cooling rate of 10 °C∙min^−1^ and followed the first heating step where the thermal processing history was removed. Figure 1b shows the melt behavior after the cooling phase. A heating rate of 10 °C∙min^−1^ was used.

As shown in Figure 1a, a tiny and barely visible crystallization peak at 101 °C was observed during the cooling of PLLA. This behavior is attributed to the slow crystallization rate of PLLA. It is well known that the higher homocrystallization capability of PLA is well established when subjected to slower cooling rates [18]. In contrast, TPE and EMA-GMA showed melt crystallization peaks at temperatures of 71 °C and 46 °C, respectively. In the case of the binary PLLA/TPE blends, a heterogeneous nucleation effect of TPE on PLLA was observed. Compared to neat PLLA (see Table 2), melt crystallization is shifted to lower temperatures (T_mc1_) and exothermic enthalpy values (ΔH_mc1_) increase with a higher TPE content. This behavior indicates an increase in nucleation density where the dispersion of the minor TPE phase acts as a nucleation site for the PLLA matrix. It also indicates that TPE partially acts as a plasticizer and shifts the melt crystallization temperature window of PLLA to lower temperatures. This behavior has been reported in several studies [90,91,92], where the expansion of the crystallization window often leads to a higher crystallinity degree of PLA. These results are consistent with the work of Samaniego et al. [93]. Their thermogravimetric analysis coupled with Fourier Transform Infrared Spectroscopy (TGA-FTIR) showed that the TPE (NPEL208) contains an “eco-friendly” plasticizer. A further decrease in melt crystallization temperature (T_mc1_) and exothermic enthalpy values (ΔH_mc1_) compared to binary counterparts (PLLA/TPE) was observed for the ternary blends after the introduction of the EMA-GMA compatibilizer. The decrease in enthalpy values indicates restricted crystallization behavior due to the addition of EMA-GMA and could be interpreted as an improvement in the compatibility of the PLLA/TPE blend. Zhang et al. [81] also observed hampered crystallization behavior of PLA/PEBA systems after the introduction of EMA-GMA. Furthermore, at a higher compatibilizer content (8.6–17.2 wt. %), secondary melt crystallization peaks (T_mc2_ and ΔH_mc2_) of EMA-GMA in ternary blends could be observed (see Table 2 and Figure 1a).

The second heating step allows for a direct comparison of the thermal behavior of neat PLLA and its uncompatibilized and compatibilized blends by excluding the previous thermal history. As can be seen in Figure 1b, the neat PLLA homopolymer exhibits a typical glass transition temperature (T_g_) at around 59 °C. A further decrease in T_g_ is observed with increasing levels of TPE and EMA-GMA (see Table 2) up to 20 wt. % and 8.6 wt. %, respectively. The glass transition temperature disappears after the introduction of minor flexible components at higher concentrations (above PLLA/20TPE/8.6EMA-GMA). The crystallinity of the PLLA phase in these blends developed during cooling to the point where the T_g_ is no longer visible. However, macromolecular crystallization often remains incomplete due to the segregation of noncrystallizable chain structures at the forefront of crystal growth, the enrichment of entanglements in the melt, and constraints in kinetics or hindered diffusion of chain segments. As a result, there is a persistent thermodynamic driving force for the continuation of the crystallization process after primary melt crystallization [94,95]. Therefore, during the heating of neat PLLA, the secondary “cold“ crystallization (T_cc_) was observed with an exothermic peak value of 99.8 °C. As a result, PLLA samples with very low crystallinity (11.7%) were obtained after cooling and subsequent heating at a rate of 10 °C∙min^−1^. For the binary blends, only PLLA/10TPE samples showed visible cold crystallization. The increased macromolecular mobility enhanced the shift in T_cc_ by almost 20 °C to a temperature of 83.9 °C. Furthermore, the exothermic enthalpy value (ΔH_cc_) dropped from 30.9 to 3.7 J∙g^−1^ when compared to neat PLLA. Consequently, the degree of crystallinity increased to a value of 36%. Furthermore, the disappearance of cold crystallization and an increase in the degree of crystallinity were observed at higher TPE contents (above 10 wt. %). As summarized in Table 2, the highest degree of crystallinity of uncompatibilized and compatibilized blends was calculated for PLLA/30TPE and PLLA/30TPE/12.9EMA-GMA with values of 46.6% and 39.4%, respectively. As previously discussed, compatibilized PLLA/TPE/EMA-GMA blends exhibited restricted primary melt crystallization when compared to binary blends. As a result, smaller cold crystallization peaks can be observed for all compatibilized blends as well as lower degrees of crystallinity (see Table 2).

Figure 1a also shows that the lower degree of crystallinity in PLLA/40TPE/17.2EMA-GMA samples compared to PLLA/30TPE/12.9EMA-GMA is due to limited conformational events of the macromolecules. This behavior is a direct result of the higher concentration of EMA-GMA, which results in a lower exothermic transformation during the primary melt crystallization. The enhancement of the PLLA/TPE interface during reactive extrusion hinders the PLLA crystallization behavior. Furthermore, two peculiarities were observed during the second DSC heating scan due to the non-isothermal cooling conditions. The first one is the emergence of a small exothermic pre-melting recrystallization peak (T_rc_) just before the primary endothermic melting peak (T_hm_) and the second one is the occurrence of a double melting peak (**T_hm_**_α′_/T_hm_). These two can be effectively elucidated by considering the crystallization conditions alongside the requirements for the formation of α′ and α crystals of PLLA in parallel. During the cooling from the relaxed melt, crystallization above 120 °C leads to the formation of α-crystals. Between 120 and 100 °C, both α- and α′-crystals can grow, and below 100 °C, only α′-crystals can develop in a neat PLLA [96,97,98]. The α′-crystals are metastable at the temperature of their formation; however, they transform into the stable α-form upon heating at around 150 °C [99,100,101]. Pre-melting recrystallization has been observed for neat PLLA, uncompatibilized samples containing up to 20 wt. % of TPE, and compatibilized samples containing up to 20 wt. % of TPE and 8.6 wt. % of EMA-GMA. However, samples with higher TPE and EMA-GMA contents exhibited double melting peaks (see Figure 1b and Table 2). Figure 1b also shows that the primary endothermic melting peaks (T_hm_) shift towards lower temperatures and the enthalpy values (ΔH_hm_) decrease with increasing TPE and EMA-GMA content. This behavior suggests partial miscibility of both TPE and EMA-GMA within PLLA. The observed plasticization in the case of PLLA/TPE may explain this phenomenon.

### 3.2. Tensile Properties

**Figure 2:**
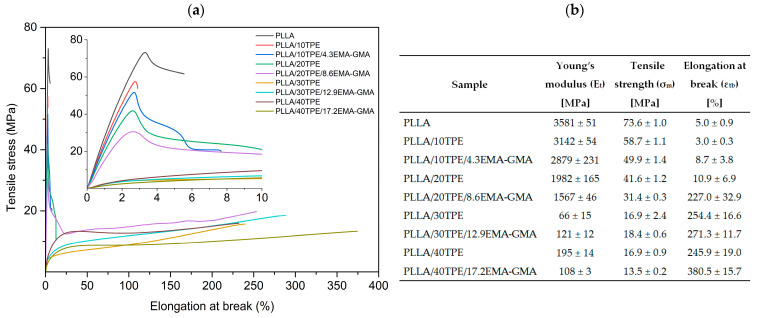
Typical examples of tensile stress-strain curves (**a**) and average tensile properties with standard deviations (**b**) of neat PLLA and both compatibilized and uncompatibilized blends. The inner graph shows detailed stress–strain curves for a smaller strain range.

Figure 2a,b present the results of tensile tests on PLLA, uncompatibilized (PLLA/TPE), and compatibilized (PLLA/TPE/EMA-GMA) blends at various concentrations. Figure 2a shows typical tensile stress–strain curves with a detailed inset for courses at small strain ranges. Figure 2b depicts the average values of the tensile modulus, tensile strength, and elongation at break with standard deviations.

The PLLA polymer is known for its brittleness, with a poor ductility of only 5%, a relatively high modulus of 3581 MPa, and strength of 73.6 MPa. When flexible TPE and EMA-GMA polymers are introduced, there is a noticeable reduction in the modulus and strength compared to neat PLLA. However, ductility does not significantly improve until the TPE and EMA-GMA composition reaches the critical concentration of 20 wt. % and 8.6 wt.%, respectively. The nominal elongation at break increased by almost 21 times when comparing PLLA/20TPE/8.6EMA-GMA samples that were compatibilized to uncompatibilized (PLLA/20TPE) ones. Samples with higher concentrations of both flexible polymers also showed distinct yielding followed by significant cold drawing, indicating a transition from brittle to ductile fracture. Moreover, a significant decrease in both tensile modulus and strength was observed in binary blends when the TPE content reached 30 wt. %. However, when samples containing 30 wt. % TPE were compatibilized with 12.9 wt. % of EMA-GMA, both the tensile modulus and strength of the ternary blends increased. In this case, the tensile modulus almost doubled when compared to the binary PLLA/30TPE blend. The given behavior indicates a transformation in the phase structure from a sea–island morphology (PLLA/30TPE) to a co-continuous one (PLLA/30TPE/12.9EMA-GMA). This same behavior, where stiffness almost doubled, was previously observed by Willemse et al. [102] when the droplet-matrix structure switched to co-continuous for PE/PS (70/30) blends. Samples based on PLLA/40TPE showed an almost threefold increase in tensile modulus (195 MPa) compared to PLLA/30TPE (66 MPa) while maintaining identical tensile strength and comparable elongation at break. The addition of a further EMA-GMA compatibilizer to the final blend (PLLA/40TPE/17.2EMA-GMA) resulted in a decrease in both the tensile modulus and strength, but a significant increase in ductility (approximately 380%). This is likely due to the inversion of co-continuous phase structures, where PLLA is a minor component (42.8 wt. %) and flexible components of the blend (TPE and EMA-GMA) serve as a base matrix material.

### 3.3. Impact Properties at Ambient and Sub-Zero Conditions

**Figure 3:**
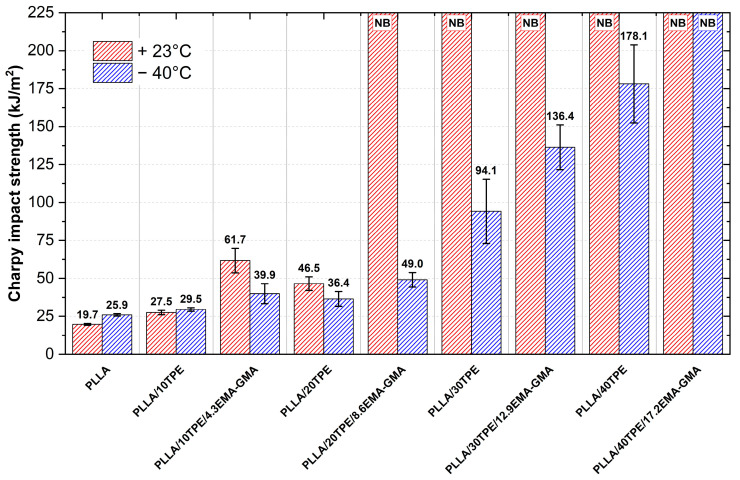
Charpy impact strength plots in ambient (23 °C) and subzero (−40 °C) conditions.

**Figure 4:**
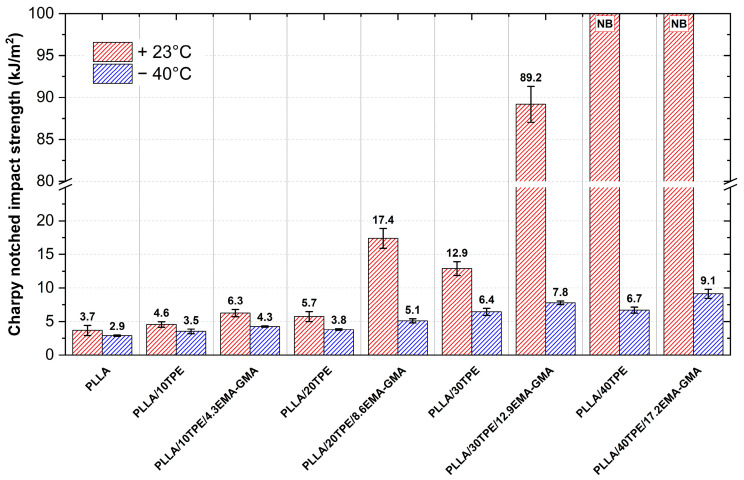
Charpy notched impact strength plots in ambient (23 °C) and subzero (−40 °C) conditions.

Neat PLLA and both uncompatibilized and compatibilized blends at various concentrations were tested for impact (Figure 3) and notched impact (Figure 4) properties at ambient (23 °C) and sub-zero (−40 °C) conditions.

As shown in Figure 3, neat PLLA exhibited typical low impact strength. It is worth noting that the impact strength was lower at ambient temperatures than in sub-zero conditions. Meekum and Khiansanoi [103] previously observed this behavior, while Mysiukiewicz et al. [104] attributed it to the intensification of the intermolecular polymer chain interactions in sub-zero conditions. The introduction of 10 wt. % of TPE resulted in only a minor improvement in impact strength, with comparable results in both conditions. However, when a compatibilizer (PLLA/10TPE/4.3EMA-GMA) was added, the impact strength was enhanced by 70% and 55% at an ambient temperature compared to neat PLLA and PLLA/10TPE, respectively. Such behavior clearly indicates synergistic improvement in rubber toughening due to enhanced interfacial adhesion at interfaces with the introduction of the EMA-GMA compatibilizer. During impact testing of PLLA/10TPE/4.3EMA-GMA samples, a decrease from 61.7 kJ∙m^−2^ at an ambient temperature to 39.9 kJ∙m^−2^ in sub-zero conditions was observed. Uncompatibilized PLLA/20TPE samples yielded medium impact strength, which predictably increased compared to PLLA/10TPE. However, when compared to compatibilized PLLA/10TPE/4.3EMA-GMA samples, the values declined. Moreover, when a blend containing 20 wt. % TPE was enhanced by 8.6 wt. % EMA-GMA, we were unable to break the resulting samples under ambient conditions. The specimens that do not break during impact testing are marked as ”NB” (Not Break) in both Figure 3 and Figure 4. As previously discussed in the tensile testing chapter, toughened ternary PLLA/20TPE/8.6EMA-GMA samples reached the brittle–ductile transition at this concentration. The typical low crack initiation energy of an amorphous and brittle PLLA matrix has been sufficiently suppressed.

However, we were able to break these samples during impact testing at −40 °C. Figure 3 shows that, beyond this critical concentration, we were unable to break any sample under ambient temperatures (using a high-energy 50 J hammer), and samples were bent below the hammer. Furthermore, a tremendous linear increase in impact strength has been observed during testing under sub-zero conditions with increasing content of both TPE and EMA-GMA. When compared to neat PLLA testing at −40 °C, 5-fold and 7-fold increases in impact strength have been observed for PLLA/30TPE/12.9EMA-GMA and PLLA/40TPE, respectively. Interestingly, when the concentration reached the final composition, we were unable to break PLLA/40TPE/17.2EMA-GMA samples even at sub-zero temperatures. Such a high level of impact strength at −40 °C is likely due to the change in the morphology of the blend to an inverted co-continuous morphology in which PLLA forms a minor phase, as demonstrated by the fracture surface study in Section 3.4.

Since we could not break most of the unnotched samples at an ambient temperature, testing of the notched impact strength has been conducted to reveal the toughening mechanisms. The results are summarized in Figure 4. Since the stress concentration has already been introduced by a sharp-tipped notch, the toughening efficiency could be observed by the increase in crack propagation energy. When compared to neat PLLA, both binary PLLA/10TPE and ternary PLLA/10TPE/4.3EMA-GMA blends showed only minor enhancement in notched impact strength. In contrast, some improvement in the prevention of fracture promotion and spreading at ambient temperature has been observed in compatibilized PLLA/20TPE/8.6EMA-GMA blends. Comparing such ternary blends to binary blends with 20 and 30 wt. % of TPE reveals the high efficiency of interphase compatibilization where 70% and 25% increases in the notched impact strength could be observed. A further influence of EMA-GMA on blend toughening can also be observed when comparing all compatibilized samples against uncompatibilized ones using impact testing under sub-zero conditions. Tremendous enhancement in the notched impact strength was achieved in the case of compatibilized PLLA/30TPE/12.9EMA-GMA blends, where an incomplete break (see graphical abstract) with a value of 89.2 kJ∙m^−2^ was observed under ambient temperature testing conditions. Such a value significantly exceeds the critical threshold of 53 kJ∙m^−2^ for labeling material with the term “super tough” [80]. The results suggest that the materials exhibited significant toughening not only under low-strain conditions (tensile testing at 5 and 1 mm∙min^−1^) but also in high-speed impact testing (an impact velocity of 3.5 m∙s^−1^). Such results support the behavior described in the tensile properties testing chapter, where a switch to a co-continuous structure was suggested. Moreover, increasing the TPE content up to 40 wt. % in binary blends and compatibilizing such blends with 17.2 wt. % EMA-GMA leads to material systems that do not break at ambient temperatures.

### 3.4. Impact Fractured Surface Morphology

**Figure 5:**
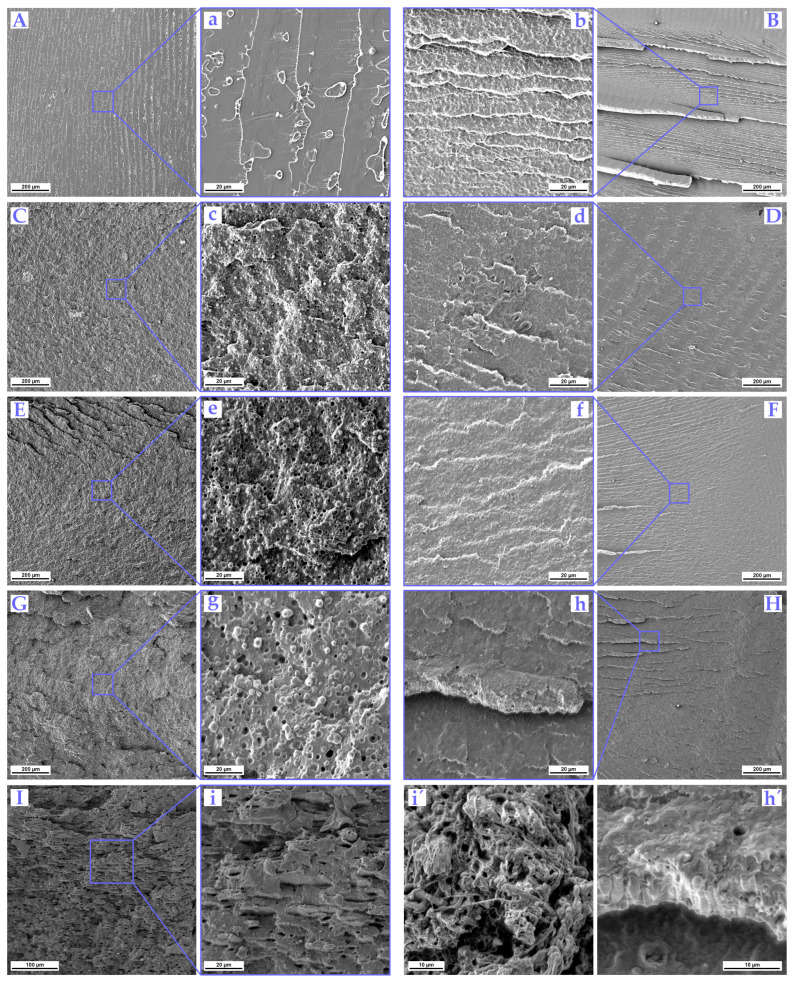
SEM images of impact cryo-fractured (−40 °C) notched samples of uncompatibilized and compatibilized blends: PLLA (**A**,**a**); PLLA/10TPE (**B**,**b**); PLLA/10TPE/4.3EMA-GMA (**C**,**c**); PLLA/20TPE (**D**,**d**); PLLA/20TPE/8.6EMA-GMA (**E**,**e**); PLLA/30TPE (**F**,**f**); PLLA/30TPE/12.9EMA-GMA (**G**,**g**); PLLA/40TPE (**H**,**h**,**h**′); PLLA/40TPE/17.2EMA-GMA (**I**,**i**,**i**′).

**Figure 6:**
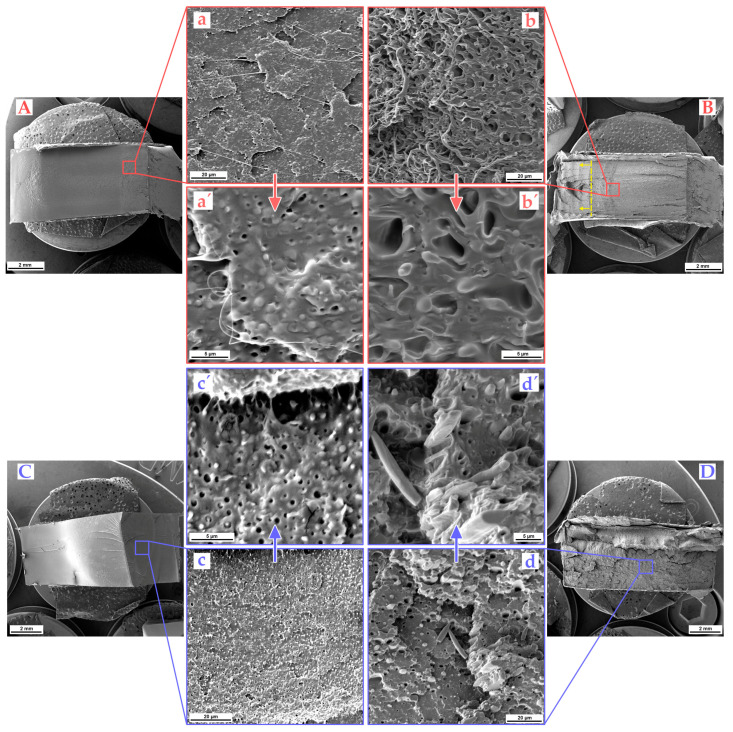
SEM images of impact-fractured surfaces of notched samples (**A**,**B**) in ambient conditions (23 °C, red) and impact cryo-fractured unnotched samples (**C**,**D**) in sub-zero (−40 °C, blue) conditions. (**A**,**a**,**a**′ and **C**,**c**,**c**′) PLLA/30TPE and (**B**,**b**,**b**′ and **D**,**d**,**d**′) PLLA/30TPE/12.9EMA-GMA.

The surface fractures of cryo-impacted specimens were studied under SEM to investigate the phase morphology of binary- and ternary-based PLLA blends. Figure 5 shows the morphology and fracture characteristics of binary and ternary blends obtained from dynamic impact loading. Since all depicted samples were notched and frozen to −40 °C overnight, typical signs of brittle polymer fracture based on crazing could be observed. Neat PLLA specimens present very smooth and brittle fractures. Figure 5A shows a faint ridged pattern on a planar surface fracture with “periodic” spacing where signs of fibrillation could be seen at higher magnification (Figure 5a). Such a pattern is known as Wallner lines, and these are formed when stress waves reflected from the specimen boundaries interact with a propagating crack front. They are typically considered snapshots of the crack front during its propagation [105]. The fracture surface of the uncompatibilized PLLA/10TPE blend sample is shown in Figure 5B. Compared to neat PLLA, the binary blend with 10 wt. % TPE exhibited a rougher surface with hackles (Figure 5B). Hackles can be seen as outward divergent lines that point along the crack propagation direction where the stress field changes rapidly in direction, magnitude, or both [106]. Crack brunching in such a way is usually the only mechanism for increasing the energy dissipation rate for brittle polymers, especially at low temperatures. From the detailed image (Figure 5b), a typical two-phase morphology of finely dispersed and distributed spherical TPE particles (seen as white dots) could be observed in the PLLA matrix. The typical sea–island phase morphology with good interfacial adhesion has been observed and the average diameter of the spherical particle size reached the value of 510 nm. Figure 5C shows a compatibilized PLLA/10TPE/4.3EMA-GMA blend sample, where a coarser and rougher failure mode was observed on fractured surfaces. This behavior suggests an increase in energy dissipation due to the creation of new surfaces during crack propagation. Furthermore, when such a system was compatibilized with 4.3 wt. % EMA-GMA, the average diameter of spherical particles increased to 1.4 μm. This trend continued as the TPE and EMA-GMA content increased to 20 and 8.6 wt. %, respectively. The average particle size reached 630 nm for PLLA/20TPE and 1.8 μm for PLLA/20TPE/8.6EMA-GMA, as shown in Figure 5d,e. This observation indicates that the EMA-GMA compatibilizer is preferably located at the PLLA/TPE interphase, where it is partly incorporated into the matrix and partly encapsulates TPE droplets. This phase separation and interphase adhesion enhancement was previously observed by Wu and Zhang [84] in the PLA/PBAT/EMA-GMA system and by Lin and Qu [43] in PLA/TPU/EMA-GMA multicomponent blends. Multicomponent systems with two minor phases also usually display core–shell-like behavior [107,108]. Furthermore, such behavior also correlates with our impact testing results. Once the TPE content reached 30 wt. %, an increase in droplet size of up to 900 nm was observed (Figure 5f). With the further introduction of a compatibilizer (PLLA/30TPE/12.9EMA-GMA), the fractured surface morphology becomes coarser (see Figure 5G) and particles become slightly distorted, increase in size (~2.4 μm), and tend to agglomerate and interconnect with each other (Figure 5g). Due to coalescence, some particles have fused and started to form fibrous structures (see Figure 6B,D). When triaxial stress concentrations reach a limit at the dispersed TPE particles, small crazes are initiated, leading to multiple-crazing behavior. Consequently, through crack propagation, new surfaces are created, and more energy is consumed. Furthermore, an increase in fractured surface roughness in PLA was also observed by Park et al. [109] and linked to a higher degree of crystallinity. This behavior is in agreement with our DSC results presented in Table 2. It is also important to note that the optimum particle size for PLA toughening varies depending on different matrix chain parameters, such as entanglement density (υ_e_) and the characteristic ratio (C_∞_), as well as its crystalline state [110]. Various experimental results suggest that the optimum particle size for high toughening efficiency falls within the range of 0.7–1.1 μm [111,112,113,114]. However, this only applies to amorphous and brittle PLA matrices, and super-tough blends can only be obtained when υ_e_ is 0.1 mmol∙cm^−3^. On the other hand, Bai et al. [114] suggest that the optimal size for a highly crystalline PLA matrix is only 0.3–0.5 μm. It is worth noting that these parameters mainly apply to binary blends and specific properties of the selected “rubber” phases. For multicomponent PLA-based blends, super toughening is typically predicted when the entanglement density is in the range of 0.12–0.14 mmol∙cm^−3^ [79]. Additionally, with an increase in TPE content up to 40 wt. %, it becomes more difficult to distinguish between PLLA and TPE phases at lower magnifications (Figure 5H). On the other hand, the detailed image in Figure 5h reveals a flattened fibrous and partially interconnected structure of the secondary TPE phase with good interphase adhesion (Figure 5h). A higher magnification image (Figure 5h′) supports the previously suggested switch to a co-continuous morphology. The last compatibilized PLLA/40TPE/17.2EMA-GMA blend can be observed in Figure 5I. Surface fracture depicts a typical co-continuous structure with a plate-like minor phase, which exhibits ductile behavior. Figure 5i shows the interconnected minor phase in detail, while Figure 5i′ provides a more magnified image of another part of the fractured surface. Additionally, a co-continuous fiber-like interconnected network phase that underwent a much larger ductile deformation is observable in Figure 5i′.

To further investigate the toughening mechanism and clarify the brittle–ductile transition behavior, we examined impact-fractured specimens of uncompatibilized PLLA/30TPE and compatibilized PLLA/30TPE/12.9EMA-GMA blends under different conditions and compared them using SEM.

Figure 6a shows that detaching TPE particles from the PLLA matrix was easier due to the notch and high-impact loading. Fractured surfaces displayed both smooth holes and the dispersion of spherical TPE particles. Despite the brittle fracture, some distorted TPE particles displayed partial fibrillation at crack tips. Additionally, the more detailed image in Figure 6a′ reveals good interfacial adhesion and cavitation of TPE domains as one of the leading energy dissipation mechanisms. Although the interphase in the sea–island phase morphology is observable, the transition between the phases is blurred. This observation is consistent with the previous statement that at least partial miscibility between TPE and PLLA likely occurred due to a shift in the glass transition temperature and melting peaks to lower values with increasing contents of both TPE and EMA-GMA (see Table 2). In the case of the notched compatibilized PLLA/30TPE/12.9EMA-GMA blend, an incomplete impact break behavior has been observed in ambient conditions (see graphical abstract). The rest of the intact sample was torn off (indicated as a dashed line with arrows) and the fracture surface can be observed in Figure 6B. Similar to the uncompatibilized blend, the fracture surface shows crack propagation in the plane due to the notch. However, the surface structure is much coarser when compared to PLLA/30TPE. In the detailed image (Figure 6b) of the first crack-arrest line, it is revealed that both the matrix and dispersed phase underwent shear yielding due to the presence of the compatibilizer. Plastic deformation is evident in the form of holes on the right side of the image where the TPE phase was torn off. On the left side, larger fibrils that have undergone plastic deformation are visible. Additionally, some fibrils have multiple connected arms, indicating the previously mentioned co-continuous phase structure. Further detail in Figure 6b′ reveals an interphase that has undergone plastic deformation, with oval holes and some small TPE droplets that have been partially deformed due to cavitation. This suggests a wider particle size distribution, where the impact stress was mainly concentrated on the large-size and compatibilized TPE phase. This observation indicates that a super-tough ternary blend, which presented ductile deformation, was achieved via the combination of shear yielding and multiple crazing.

Figure 6C,D provide a comparison of the fractured surfaces of PLLA/30TPE and PLLA/30TPE/12.9EMA-GMA blends. However, these are unnotched and were broken at sub-zero temperatures (−40 °C). A general view of the fracture surface of PLLA/30TPE in Figure 6C shows a brittle failure with a smooth fracture surface and several hackles. The detailed image in Figure 6c shows the dispersion of spherical TPE particles and holes where the advancing crack front has pulled out TPE. As demonstrated in the detailed images (Figure 6c,c′), cavitation is the primary energy dissipation mechanism of unnotched binary samples during fracture at −40 °C. A comparison of these fractured surfaces with those of binary blend samples, which were notched and fractured at 23 °C, reveals that the unnotched samples broken at sub-zero temperatures exhibit significantly more cavitation and plastic deformation of TPE domains (see Figure 6c′). Although the PLLA matrix is below its glass transition temperature, the TPE remains in a rubbery state. As a result, spherical particles deform and debond easily at −40 °C. The results indicate that the addition of 30 wt. % of TPE greatly enhanced the low crack initiation energy of neat PLLA even in sub-zero testing conditions, as shown in Figure 3. The sub-zero fracture of the unnotched and compatibilized ternary blend can be seen in Figure 6D. A view of the total fracture surface of the PLLA/30TPE/12.9EMA-GMA specimen exhibits a very coarse failure structure with deep cracks, indicating crack propagation in multiple directions. This behavior indicates massive impact energy absorption through the generation of new surfaces during fracture. In contrast to the binary blend, which exhibits a sea–island morphology, the compatibilized blends at this TPE loading, with the assistance of EMA-GMA, form a fiber-like morphology structure within the PLLA matrix during sample processing, as shown in the detailed image in Figure 6d. The fibrous structure is oriented perpendicular to the direction of impact stress and exhibits excellent interfacial adhesion. Unlike notched samples that broke at an ambient temperature, the fractured surface did not exhibit a co-continuous structure. Some fibers on the fracture surface displayed typical necking and tapering at the point of failure, while others exhibited brittle failure (refer to Figure 6d′). The detailed picture reveals debonding along the fiber direction during crack propagation. Based on these results, it can be speculated that EMA-GMA acted as a bridge between the PLLA/TPE phases, resulting in high interfacial adhesion. Such a high interfacial adhesion resulted in failure where it was impossible to reveal a co-continuous structure because the fibers prematurely broke due to high-impact loading at low testing temperatures. Liu et al. [115] found that debonding followed by matrix shear yielding is a much more significant toughening mechanism than the internal cavitation of rubber particles. They observed delayed shear yielding of the matrix in poly(vinyl chloride) (PVC)/nitrile rubber(NBR) blends with high acrylonitrile (AN) content (26%). The premature break was caused by the absence of debonding at interphase and microvoid formation.

## 4. Conclusions

In this work, the inherent brittleness of poly(l-lactic acid) (PLLA) has been addressed by melt blending with the biodegradable thermoplastic polyester elastomer (TPE) and a compatibilizer based on an ethylene-methyl acrylate-glycidyl methacrylate (EMA-GMA) copolymer. Differential scanning calorimetry (DSC) analysis revealed that the TPE phase works as a nucleation site for the matrix and increases the crystallinity degree of PLLA. Furthermore, the increasing content of TPE and EMA-GMA causes a shift in the melt crystallization, glass transition, and melting temperatures of PLLA to lower values. This suggests that the two components are at least partially miscible. Typically, a decrease in tensile modulus and tensile strength has been observed with an increasing content of both flexible polymers. The brittle–ductile transition, with a dramatic increase in elongation at break, was detected when the PLLA/20TPE blend was compatibilized by 8.6 wt. % of EMA-GMA. We could not break any unnotched binary and ternary samples from this concentration and above at room temperature. Furthermore, impact testing of unnotched samples at sub-zero conditions (−40 °C) revealed increased enhancement in crack initiation resistance with increasing TPE and EMA-GMA contents. For instance, the unnotched PLLA/40TPE blend showed an impressive sub-zero impact strength of 178.1 kJ∙m^−2^. Impact testing of notched samples confirmed the significant influence of the compatibilizer on blend toughening and crack propagation resistance in both ambient and sub-zero conditions. The “super tough” behavior of the PLLA/30TPE/12.9EMA-GMA ternary blend with the incomplete break and notched impact strength of 89.2 kJ∙m^−2^ has been observed at an ambient temperature (23 °C). Furthermore, we could not break notched PLLA/40TPE and PLLA/40TPE/17.2EMA-GMA samples at an ambient temperature. Morphological observation by scanning electron microscopy (SEM) confirms good interfacial adhesion between PLLA and TPE. Observations also indicate that the EMA-GMA is preferably located at the PLLA/TPE interphase, where it is partially incorporated into the matrix and partially encapsulates TPE. SEM also confirms that the transition from sea–island to co-continuous morphology is responsible for the super tough behavior. We believe that a combination of good interfacial adhesion, debonding cavitation, and subsequent matrix shear yielding worked synergistically with the transition from sea–island to co-continuous phase morphology to form an excellent super-toughening mechanism.

## Figures and Tables

**Table 1:** Composition of the blends.

Sample	PLLA (wt. %)	TPE (wt. %)	EMA-GMA (wt. %)
PLLA	100	0	0
PLLA/10TPE	90	10	0
PLLA/10TPE/4.3EMA-GMA	85.7	10	4.3
PLLA/20TPE	80	20	0
PLLA/20TPE/8.6EMA-GMA	71.4	20	8.6
PLLA/30TPE	70	30	0
PLLA/30TPE/12.9EMA-GMA	57.1	30	12.9
PLLA/40TPE	60	40	0
PLLA/40TPE/17.2EMA-GMA	42.8	40	17.2

**Table 2:** Thermal parameters obtained from DSC analysis during the cooling and subsequent heating at a rate of 10 °C∙min^−1^ and crystallinity degrees of the PLLA phase.

Sample	Cooling				2nd Heating							
	T_mc1_	ΔH_mc1_	T_mc2_	ΔH_mc2_	T_g_	T_cc_	ΔH_cc_	T_rc_ (T_hmα′_)	ΔH_rc_	T_hm_	ΔH_hm_	χ_c_
	[°C]	[J∙g^−1^]	[°C]	[J∙g^−1^]	[°C]	[°C]	[J∙g^−1^]	[°C]	[J∙g^−1^]	[°C]	[J∙g^−1^]	[%]
PLLA	101.1	1.2	-	-	59.4	99.8	30.9	156.9	6.2	173.0	49.5	11.7
PLLA/10TPE	97.0	23.3	-	-	48.4	83.9	3.7	153.4	4.1	172.1	42.2	36.0
PLLA/10TPE/4.3EMA-GMA	95.6	16.0	-	-	49.7	83.8	7.8	151.9	5.3	171.4	40.3	30.0
PLLA/20TPE	93.5	26.1	-	-	38.5	-	-	153.9	2.2	170.5	34.9	38.5
PLLA/20TPE/8.6EMA-GMA	89.8	17.9	46.6	1.8	36.5	71.1	1.6	149.9	3.0	168.9	33.2	37.8
PLLA/30TPE	96.7	28.2	-	-	-	-	-	(154.0)	-	166.9	34.6	46.6
PLLA/30TPE/12.9EMA-GMA	90.2	20.6	46.7	2.1	-	84.6	3.0	(152.9)	-	167.0	26.9	39.4
PLLA/40TPE	97.1	24.7	-	-	-	-	-	(151.4)	-	165.0	28.6	44.9
PLLA/40TPE/17.2EMA-GMA	91.8	16.9	46.6	2.8	-	81.9	3.2	(152.9)	-	165.5	19.6	36.1

## Data Availability

The data presented in this study are available upon request from the corresponding author.
